# Grouper as a Natural Biocontrol of Invasive Lionfish

**DOI:** 10.1371/journal.pone.0021510

**Published:** 2011-06-23

**Authors:** Peter J. Mumby, Alastair R. Harborne, Daniel R. Brumbaugh

**Affiliations:** 1 Marine Spatial Ecology Lab, School of Biological Sciences, University of Queensland, St. Lucia, Queensland, Australia; 2 Biosciences, Hatherly Laboratory, College of Life and Environmental Sciences, University of Exeter, Exeter, Devon, United Kingdom; 3 Center for Biodiversity and Conservation, American Museum of Natural History, New York, New York, United States of America; 4 Institute of Marine Sciences, University of California Santa Cruz, Santa Cruz, California, United States of America; Smithsonian's National Zoological Park, United States of America

## Abstract

Lionfish (*Pterois volitans/miles*) have invaded the majority of the Caribbean region within five years. As voracious predators of native fishes with a broad habitat distribution, lionfish are poised to cause an unprecedented disruption to coral reef diversity and function. Controls of lionfish densities within its native range are poorly understood, but they have been recorded in the stomachs of large-bodied Caribbean groupers. Whether grouper predation of lionfish is sufficient to act as a biocontrol of the invasive species is unknown, but pest biocontrol by predatory fishes has been reported in other ecosystems. Groupers were surveyed along a chain of Bahamian reefs, including one of the region's most successful marine reserves which supports the top one percentile of Caribbean grouper biomass. Lionfish biomass exhibited a 7-fold and non-linear reduction in relation to the biomass of grouper. While Caribbean grouper appear to be a biocontrol of invasive lionfish, the overexploitation of their populations by fishers, means that their median biomass on Caribbean reefs is an order of magnitude less than in our study. Thus, chronic overfishing will probably prevent natural biocontrol of lionfishes in the Caribbean.

## Introduction

Over the last five years, one of the world's most ornate fishes, the lionfish (*Pterios volitans/miles*), has invaded much of the Caribbean, spanning an area exceeding 5,000 km^2^ [1]. It is generally believed that *P. volitans*, together with a sister species, *P. miles*, escaped from aquaria in Florida within the last decade [2]. What makes the invasion of these species so important is their voracious appetite for small fishes [3], [4], [5], combined with their ability to invade multiple habitats, ranging from the outer margins of reefs to sheltered mangrove lagoons [6], [7]. Thus, small-bodied and juvenile reef fish are now subjected to greatly elevated predation and the usual strategies employed to avoid predation, such as the usage of mangrove nurseries [8], [9], may confer limited benefit as lionfish occupy most habitats. The long-term consequences of such predation on reef biodiversity and function are not yet clear, but are a matter of grave concern.

The success of lionfish is partly attributable to its resistance to predation, largely because of its elaborate portfolio of venomous spines. In its native range of the Indo-Pacific, identification of the predators of lionfish has proven elusive, although reports of predation by the cornetfish, *Fistularia commersoni*, have been made for *P. miles* [10]. Fistularids are uncommon in the Caribbean [11] and the only confirmed indication of predation has been the observation of lionfishes in the stomachs of two large-bodied species of grouper, *Epinephelus striatus* and *Myceteroperca tigris* [12]. Here, we ask whether grouper, one of the most heavily targeted fisheries species in the world [13], could serve as a natural form of biocontrol for invasive lionfishes. The use of predatory fishes for controlling invasive species has been used in other ecosystems [14], but has not previously been described for coral reefs.

While grouper may have the capacity to consume lionfish, this does not necessarily imply that they act as a biocontrol. Effective biocontrol would require that grouper predation exerted a significant net impact on the density of their prey, which might not be the case if predation rates are low and/or lionfish recruitment rates high. To establish whether grouper represent a natural form of biocontrol for lionfish, we took advantage of a natural fishing experiment in the Bahamas. A 20 year ban on fishing within the Exuma Cays Land and Sea Park (ECLSP) has enabled grouper populations to exceed those in fished areas [15]. Some five years after the invasion began, we ask whether these increased densities of groupers are reducing lionfish densities and, if so, consider whether this biocontrol mechanism is likely to be feasible elsewhere in the Caribbean region.

## Results and Discussion

**Figure 1:**
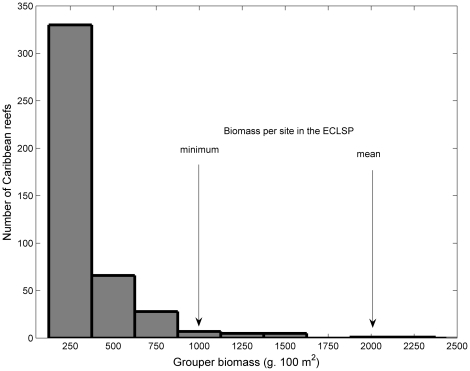
Frequency distribution of the number of sites supporting the current range of grouper biomasses recorded in the Caribbean. Data derived from the Atlantic Gulf Rapid Reef Assessment database. ECLSP  =  Exuma Cays Land and Sea Park.

A 20 year ban on fishing in the Exuma Cays Land and Sea Park (ECLSP) has allowed predatory groupers to attain some of the highest biomasses reported anywhere in the Caribbean. Taking the region-wide dataset from the Atlantic Gulf Rapid Reef Assessment Program [16], we show that the mean biomass of grouper in the Park, ∼2000 g 100 m^−2^, falls in the top 1% of all Caribbean sites (Fig. 1). Thus the ECLSP and it's surrounding areas provide an unusual opportunity to examine the potential of groupers to act as a natural biocontrol of non-native lionfish.

**Figure 2:**
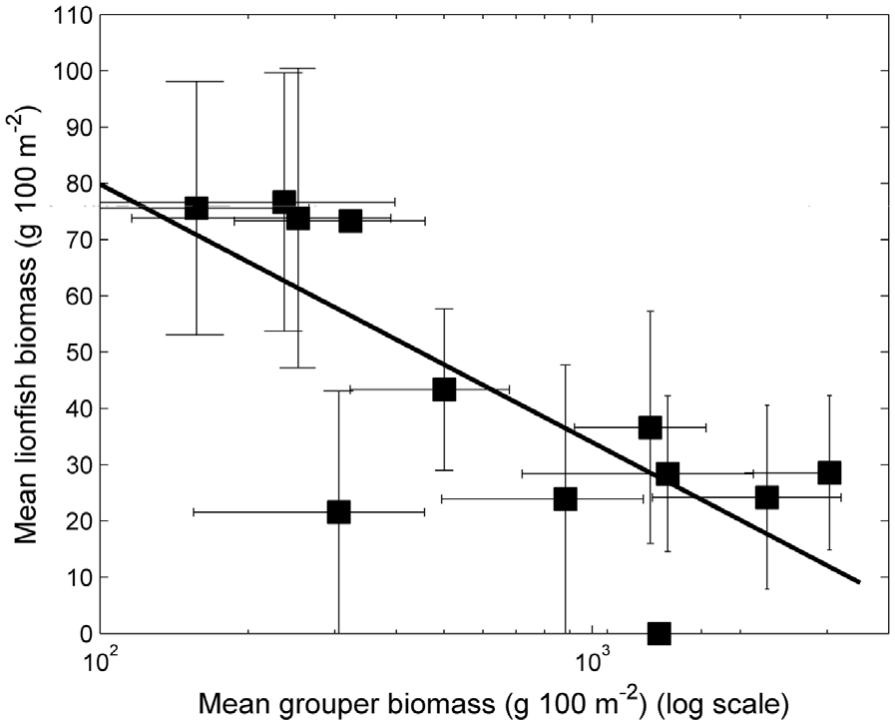
Relationship between grouper biomass and lionfish density. Open squares are sites outside the Exuma Cays Land and Sea Park, filled squares are sites within the Park. Errors bars represent standard error.

The biomass of lionfish was significantly negatively correlated with the biomass of grouper, with predator biomass explaining 56% of the variance of prey biomass (linear regression p = 0.005, Fig. 2, Table 1). Unlike large-bodied groupers (mean total length 55 cm, range 30–110 cm), other smaller predatory fishes such as *Cephalopholis* spp., lutjanids, carangids and aulostomids had no significant bearing on lionfish biomass (p = 0.17, Table 1), which might imply that large-bodied fish are the primary predators of lionfish. The relationship of grouper on lionfish was strongly non-linear such that an 18-fold variation in predator biomass among sites (∼170–3000 g 100 m^−2^) was related to a tenfold difference in lionfish density (∼0.3–0.03 fish 100 m^−2^) and 7-fold difference in lionfish biomass (Fig. 2). A 50% reduction in lionfish biomass was achieved with a grouper biomass of 800 g 100 m^−2^. Reducing lionfish density to 30% its highest value required a further doubling of grouper biomass to approximately 1516 g 100 m^−2^ (Fig. 2). The mean body length of lionfish was 24.5 cm (SD 4.1, range 15–34 cm).

**Table 1:** Linear regression of lionfish biomass (g 100 m^−2^) on the biomass of grouper (g 100 m^−2^) and other predator fishes (g 100 m^−2^).

Predictor	Coefficient	Std. Dev.	T	p
Constant	224.34	45.41	4.94	0.001
Grouper biomass (log)	−59.54	15.30	−3.89	0.004
Other predator biomass	0.008	0.005	1.46	0.179

R^2^ = 0.56. Std. Dev. denotes standard deviation.

Biocontrol is defined as the use of living organisms to suppress the population density or impact of a specific pest organism, making it less abundant or less damaging than it would otherwise be [17]. Thus, at high levels, grouper appear to be a natural biocontrol of lionfish, and likely caused the 7-fold difference in lionfish biomass within a 30 km stretch of reef. Although we have no data on whether this biocontrol is driven by grouper predation or competitive pressure, three lines of evidence suggest that predation is the most likely mechanism. First, lionfish have been found in grouper stomachs [12] so there is direct evidence supporting the mechanism. Second, large-bodied species of grouper, such as *Epinephelus striatus*, are unlikely to share the same food resources as lionfish. A study comparing predation by small individuals of *E. striatus* and a meso-predator that feeds on similar-sized prey to lionfish [4], [5], *Cephalopholis fulva*, found that the large-bodied grouper species targeted larger food items [18] such as fish [19]. Given that the large-bodied grouper in our study were mostly adults and larger than those studied by Stallings [18], it seems even more unlikely that the two groups compete for food. Third, studies of interactions between adult grouper and smaller, reef meso-predators have discovered predation-based behavioural responses of the prey rather than evidence of direct competition [20].

There remains much to learn about the scope for biocontrol of lionfish. Laboratory and field trials are needed to understand the size-dependency of the predator-prey relationship and the role that small-bodied grouper and other piscivores may play, particularly in preying upon juvenile lionfish. We also observed that lionfish appeared to remain closer to refugia at sites with high grouper densities suggesting that grouper may both reduce lionfish densities and reduce the predation rates of lionfish in the area, as they do for functionally similar small-bodied grouper [20]. Given that lionfish were absent in 2006 and only began to appear throughout the Exumas in 2007, our results suggest the ability of groupers to constrain the invasion over a 3 year period. It is feasible that the absolute density of lionfish may change as the invasion continues and repeated monitoring will study any change in the relationship with grouper biomass. Further, it is not clear whether continued recruitment of lionfish in non-reefal habitats, that lack large predatory grouper [6], might increase the future settlement of lionfish on reefs and challenge the efficacy of biocontrol in grouper habitats. Finally, it is unclear whether sharks play an active role in lionfish predation. The entire Exuma Cays possess relatively high densities of reef sharks, with at least one individual encountered per hour at each site. However, while sharks are unlikely to have contributed to the gradients of lionfish density observed here - because they were found throughout the study region and are protected from fishing throughout the bahamas - it is possible that they depress the overall density of lionfish in The Bahamas [3].

Although the impacts of the lionfish invasion on biodiversity and ecosystem function are not yet clear, our study provides insights into the feasibility of natural biocontrol. It is sobering that overfishing of large-bodied grouper [21], [22] has left the median biomass at only 178 g 100 m^−2^ in the Caribbean (Figure 1). Our results suggest that such a low biomass, which is more than an order of magnitude less than that in the ECLSP, would have only a minor impact on lionfish biomass because it is barely detectable using our regression relationship. Thus, while grouper appear to have the capacity to serve as a natural biocontrol, this is only likely to be feasible in protected marine reserves [23] or if fishing practices change to allow the maintenance of large individuals in the population. One potential option would be the usage of slot fisheries, that target intermediate-sized fishes and leave the largest individuals to maintain reproduction and ecosystem processes [24]. However, if the historical trend of poor management continues [25] then direct capture and eradication may be the only practicable form of lionfish control for much of the Caribbean. Economic incentives, such as the development of a commercial market for lionfish - which is beginning to happen in some parts of the Caribbean - might help make direct interventions a cost-effective option.

## Materials and Methods

In May 2010, 12 sites were selected along a stretch of reef spanning 30 km of the Exuma Cays, central Bahamas (Table S1). Five sites were located in the ECLSP and seven sites were located in fished areas to the north of the Park. Eight of the sites had been visited before the invasion [26] and were therefore revisited. Additional sites were selected near mooring buoys that provided the same *Montastraea* reef habitat at a suitable depth of 7–15 m. No lionfish were seen at any site in 2004 or 2006. In May 2007, a survey of nine sites (six of the sites chosen in 2010, plus three to the south of the Park) recorded the presence of lionfish both to the north and south of the Park, though densities were too low for effective fish censuses. Dive boats had not collected lionfish at dive sites in the Exuma Cays for at least 2 years prior to surveys. Indeed, the highest and lowest densities of lionfish were both observed at dive sites that receive occasional visitation (once per week).

The same habitat (complex *Montastraea* forereef at a depth of 7–15 m) was surveyed at each site. This is the preferred habitat of adult large-bodied grouper [27]. Previous studies along the Exuma Cays found no systematic variation across reserve boundaries in habitat complexity or predicted fish larval supply [28]. Lionfish and all large-bodied species of grouper (*Epinephelus striatus*, *Mycteroperca tigris*, *M. bonaci*, *M. venenosa,* and *M. interstitialis*; subsequently ‘grouper’) were visually censused simultaneously using eight replicate timed swims per site. Timed swims represent a more appropriate and efficient technique for censusing relatively rare and patchy species than more traditional transect approaches. Each timed swim lasted 5 minutes, covered approximately 300 m^2^, and was undertaken by the same surveyor (PJM). Care was taken to examine cryptic habitats and the count, size and species of individuals was recorded. Additional predatory fishes in the families Sphryaenidae, Serranidae, Lutjanidae, Aulostomidae, and Muraenidae were sampled using eight 30×4 m transects per site (Muraenidae have been observed to consume a wounded lionfish [7]). Given that biomass is a better proxy for trophic impact than density, grouper data were converted to mass using allometric scaling relationships with body length [29]. Allometric scaling relationships for lionfish were obtained elsewhere [30].

To assess patterns of grouper biomass around the Caribbean and place the Exuma Cays data in context, we extracted data from 443 forereef sites in the Atlantic Gulf Rapid Reef Assessment (AGRRA) database [16]. Sites spanned 15 countries including the British Virgin Islands, US Virgin Islands, Florida, Venezuela, Puerto Rico, Panama, Bonaire, Curaçao, Mexico, Cayman Islands, Cuba, Costa Rica, Belize, Jamaica and The Bahamas. These data were collected using visual fish census along 30×4 m transects. In order to compare grouper biomass from our Exumas sites to the AGRRA data, we also measured grouper biomass along nine 30×4 m transects at nine of our sites in 2010 and five 50×4 m transects in 2004. Surveys in 2007 and 2004 also quantified the total fish community structure for non-cryptic species.

## Supporting Information

Table S1 Survey locations in the Exuma Cays. Reserve status denotes whether the site was within the Exuma Cays Land and Sea Park.
(DOC)
