# Supplementary material for: Grouper as a Natural Biocontrol of Invasive Lionfish
Source: PLoS One. 2011 Jun 23;6(6):e21510. doi: 10.1371/journal.pone.0021510 (PMC3121772; doi:10.1371/journal.pone.0021510)
Supplement: Table S1 — Survey locations in the Exuma Cays. Reserve status denotes whether the site was within the Exuma Cays Land and Sea Park. (DOC) [file pone.0021510.s001.doc]

Table S1. Survey locations in the Exuma Cays. Reserve status denotes whether the site was within the Exuma Cays Land and Sea Park.

| Site Number | Northing | Easting | Reserve status | Depth (m) |
| --- | --- | --- | --- | --- |
| 1 | 2737168 | 317485 | No | 15 |
| 2 | 2726350 | 318330 | No | 9 |
| 3 | 2724947 | 318590 | No | 10 |
| 4 | 2702475 | 330172 | Yes | 10 |
| 5 | 2703067 | 329103 | Yes | 13 |
| 6 | 2704791 | 326517 | Yes | 14 |
| 7 | 2702817 | 329182 | Yes | 11 |
| 8 | 2711648 | 322963 | Yes | 7 |
| 9 | 2731390 | 317795 | No | 15 |
| 10 | 2731810 | 317781 | No | 17 |
| 11 | 2741773 | 316502 | No | 10 |
| 12 | 2737472 | 317386 | No | 12 |
